# Risk of Serious Infection in Patients with Psoriasis Receiving Biologic Therapies: A Prospective Cohort Study from the British Association of Dermatologists Biologic Interventions Register (BADBIR)

**DOI:** 10.1016/j.jid.2017.10.005

**Published:** 2018-03

**Authors:** Zenas Z.N. Yiu, Catherine H. Smith, Darren M. Ashcroft, Mark Lunt, Shernaz Walton, Ruth Murphy, Nick J. Reynolds, Anthony D. Ormerod, Christopher E.M. Griffiths, Richard B. Warren, BADBIR Study Group

**Affiliations:** 1 Dermatology Centre, Salford Royal NHS Foundation Trust, The University of Manchester, Manchester Academic Health Science Centre, NIHR Manchester Biomedical Research Centre, Manchester, UK; 2 Centre for Pharmacoepidemiology and Drug Safety, School of Health Sciences, The University of Manchester, Manchester, UK; 3 St. John’s Institute of Dermatology, Guy’s and St. Thomas’ NHS Foundation Trust, London, UK; 4 Arthritis Research UK Epidemiology unit, The University of Manchester, Manchester, UK; 5 Department of Dermatology, Castle Hill Hospital, Hull, UK; 6 Sheffield University Teaching Hospitals and Sheffield Children’s Hospitals, Sheffield, UK; 7 Dermatological Sciences, Institute of Cellular Medicine, Medical School, Newcastle University, and Department of Dermatology, Royal Victoria Infirmary, Newcastle Hospitals NHS Foundation Trust, Newcastle upon Tyne, UK; 8 Division of Applied Medicine, University of Aberdeen, Foresterhill, Aberdeen, UK

**Keywords:** BADBIR, British Association of Dermatologists Biologic Interventions Register, CI, confidence interval, HR, hazard ratio, IQR, interquartile range, PSOLAR, Psoriasis Longitudinal Assessment and Registry, TNFI, tumor necrosis factor inhibitor

## Abstract

Serious infection is a concern for patients with psoriasis receiving biologic therapies. We assessed the risk of serious infections for biologics used to treat psoriasis by comparison with a cohort receiving non-biologic systemic therapies in a propensity score-weighted Cox proportional hazards model using data from the British Association of Dermatologists Biologic Interventions Register. Overall, 1,352; 3,271; and 994 participants were included in the etanercept, adalimumab, ustekinumab cohorts, respectively, and 3,421 participants were in the non-biologic cohort. A total of 283 patients had a serious infection; the incidence rates with 95% confidence intervals (CI) per 1,000 person-years were as follows: non-biologic, 14.2 (11.5–17.4); etanercept, 15.3 (11.6–20.1); adalimumab, 13.9 (11.4–16.6); and ustekinumab, 15.1 (10.8–21.1). No significant increases in the risk of serious infection were observed for etanercept (hazard ratio [HR] = 1.10, 95% CI = 0.75–1.60), adalimumab (HR = 0.93, 95% CI = 0.69–1.26), or ustekinumab (HR = 0.92, 95% CI = 0.60–1.41) compared with non-biologic systemic therapies or methotrexate-only (etanercept: HR = 1.47, 95% CI = 0.95–2.28; adalimumab: HR = 1.26, 95% CI = 0.86–1.84; ustekinumab: HR = 1.22, 95% CI = 0.75–1.99). The risk of serious infection should not be a key discriminator for patients and clinicians when choosing between non-biologic systemic therapies, etanercept, adalimumab, and ustekinumab for the treatment of psoriasis.

## Introduction

Moderate to severe psoriasis is increasingly managed by biologic, immune-modulating therapies. The main adverse event leading to discontinuation of biologic therapies in patients with psoriasis is infection (Warren et al., 2015). Serious infections are associated with significant morbidity or mortality. Thus, patients considering the switch from less effective non-biologic systemic therapies to biologic therapies are concerned about whether these treatments are associated with a greater risk of serious infections.

The risk of serious infection for biologic therapies in the real world has been hard to ascertain, because clinical trials have limited external validity (Garcia-Doval et al., 2012) and are not powered to assess this outcome (Yiu et al., 2016). Currently, the risk of serious infections in patients with psoriasis on biologic therapies is not well-quantified. Three prospective observational cohort studies, using different methodologies and different comparators, have unsurprisingly reported conflicting results (Davila-Seijo et al., 2017, Garcia-Doval et al., 2017a, Kalb et al., 2015). One showed no increased risk of serious infection with tumor necrosis factor inhibitors (TNFIs) compared with acitretin, methotrexate, or cyclosporine (Garcia-Doval et al., 2017b), and the two other studies showed no significant increased risk with TNFIs or ustekinumab versus methotrexate (Davila-Seijo et al., 2017) and an increased risk with adalimumab and infliximab compared with retinoids/phototherapy (Kalb et al., 2015). However, these studies have different specific limitations—for example, a smaller sample size and lack of power to investigate serious infections (Davila-Seijo et al., 2017), limited analytical methodology in adjusting for potential confounders (Kalb et al., 2015), and inclusion of TNFIs only (Garcia-Doval et al., 2017a)—which may have accounted for the contradictory results.

Our objective was to determine whether the biologic therapies recommended by the National Institute for Health and Care Excellence in the UK for patients with moderate to severe psoriasis (defined as Psoriasis Area and Severity Index of at least 10 and Dermatology Life Quality Index of more than 10)—etanercept, adalimumab, or ustekinumab—elevate the risk of serious infection more than non-biologic systemic therapies in patients with psoriasis. To address this, we used a large, national, prospective psoriasis registry, the British Association of Dermatologists Biologic Interventions Register (BADBIR).

## Results

**Figure 1:**
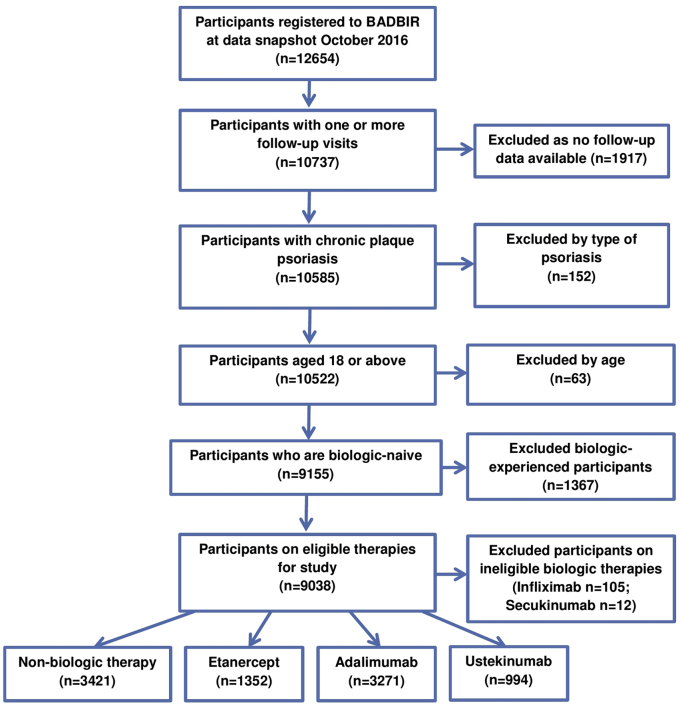
**Participant inclusion and exclusion flow diagram.** BADBIR, British Association of Dermatologists Biologic Interventions Register.

A total of 9,038 participants were included for analysis (Figure 1), with 3,421 participants included in the non-biologic systemic cohort and 5,617 participants included in the biologic cohort through October 2016. The number of participants lost to follow-up at each data collection point is listed in Supplementary Table S1 online. The baseline demographic, anthropometric, and disease characteristics of the participants are listed in Table 1. The total and median follow-up times for the biologic therapies were 13,369.81 and 1.95 person-years (interquartile range [IQR] = 2.24 person-years), respectively. Adalimumab had the longest total person-time follow-up of 7,835.17 person-years and ustekinumab the shortest, at 2,256.44 person-years (Table 2). The total person-time follow-up and median follow-up times for the non-biologic cohort were 6,419.24 person-years and 1.51 (IQR = 1.84 person-years) person-years, respectively.

**Table 1:** The baseline demographic and disease characteristics of the study cohort

Patient Characteristics	Non-biologic cohort (n = 3,421)	Etanercept (n = 1,352)	Adalimumab (n = 3,271)	Ustekinumab (n = 994)
Demographics				
Age in years, mean (SD)	44.6 (14.0)	45.5 (12.9)	44.7 (12.5)	45.9 (13.2)
Female	1489 (43.5)	565 (41.8)	1323 (40.4)	377 (37.9)
Waist circumference in cm, mean (SD)	99.7 (17.1)	101.7 (16.6)	101.2 (16.8)	104.7 (19.0)
BMI category, n (%)				
Underweight (<18.5 kg/m^2^)	43 (1.3)	17 (1.3)	22 (0.7)	8 (0.8)
Normal (18.5–24.9 kg/m^2^)	677 (19.8)	236 (17.5)	546 (16.7)	149 (15.0)
Overweight (25.0–29.9 kg/m^2^)	1071 (31.3)	408 (30.2)	1031 (31.5)	262 (26.4)
Obese I (30.0–34.9 kg/m^2^)	735 (21.5)	296 (21.9)	787 (24.1)	211 (21.2)
Obese II (35.0–39.9 kg/m^2^)	345 (10.1)	178 (13.2)	381 (11.6)	141 (14.2)
Obese III (≥40 kg/m^2^)	279 (8.2)	107 (7.9)	275 (8.4)	141 (14.2)
Comorbidities and risk factors^1^				
No comorbidity	1323 (38.7)	377 (27.9)	1016 (31.1)	323 (32.5)
1–2 comorbidities	1585 (46.3)	689 (51.0)	1636 (50.0)	436 (43.9)
3–4 comorbidities	416 (12.2)	233 (17.2)	513 (15.7)	178 (17.9)
≥5 comorbidities	97 (2.8)	53 (3.9)	106 (3.2)	57 (5.7)
Hypertension	620 (18.1)	390 (28.8)	782 (23.9)	259 (26.1)
Past TB	21 (0.6)	22 (1.6)	29 (0.9)	8 (0.8)
Diabetes mellitus	254 (7.4)	135 (10.0)	251 (7.7)	112 (11.3)
Dyslipidemia	307 (9.0)	143 (10.6)	334 (10.2)	120 (12.1)
Asthma	361 (10.6)	137 (10.1)	340 (10.4)	120 (12.1)
COPD	69 (2.0)	17 (1.3)	45 (1.4)	26 (2.6)
Immunodeficiency syndromes	6 (0.2)	4 (0.3)	1 (0.0)	4 (0.4)
Number of cigarettes smoked per day, mean (SD)	4.6 (7.7)	4.0 (7.1)	3.8 (6.8)	3.8 (7.1)
Alcohol units per week, mean (SD)	7.7 (12.1)	9.4 (17.1)	8.4 (13.4)	7.7 (12.8)
Disease				
Disease duration in years, median (IQR)	18.0 (18.0)	21.0 (18.0)	20.0 (17.0)	20.0 (19.0)
Baseline PASI score, median (IQR)	14.1 (7.9)	13.8 (7.8)	14.3 (8.6)	14.6 (8.1)
Inflammatory arthritis	363 (10.6)	316 (23.4)	819 (25.0)	158 (15.9)
Concomitant treatments, n (%)				
Any exposure to methotrexate during follow-up	2,118 (61.9)	229 (16.9)	572 (17.5)	83 (8.4)
Any exposure to cyclosporine during follow-up	1,216 (35.6)	104 (7.7)	225 (6.9)	38 (3.8)
Any exposure to acitretin during follow-up	970 (28.4)	48 (3.6)	71 (2.2)	27 (2.7)
Any exposure to fumaric acid esters during follow-up	552 (16.1)	26 (1.9)	30 (0.9)	5 (0.5)
Any exposure to hydroxycarbamide during follow-up	56 (1.6)	6 (0.4)	17 (0.5)	10 (1.0)

Abbreviations: BMI, body mass index; COPD, chronic obstructive pulmonary disease; IQR, interquartile range; PASI, Psoriasis Area and Severity Score; SD, standard deviation; TB, tuberculosis.

1 List of predefined comorbidities includes hypertension, angina, myocardial infarction, stroke, epilepsy, asthma, chronic obstructive pulmonary disease, peptic ulcer disease, chronic renal disease, liver disease, previous tuberculosis, demyelination, diabetes mellitus, impaired glucose tolerance, depression, dyslipidemia, nonskin cancer, immunodeficiency syndromes, and thyroid disease.

**Table 2:** Crude incidence rates of first serious infection: overall, lower respiratory tract infections, skin and soft tissue infections

Treatment (n)	Total Person-Time, (Median Follow-Up Time) in Years	Infections	Rate per 1,000 Person-Years	95% Confidence Interval in Person-Years
All serious infections				
Non-biologics (3,421)	6,419.24 (1.51)	91	14.18	11.54–17.41
Methotrexate (2,118)	3,422.40 (1.27)	41	11.98	8.82–16.27
Etanercept (1,352)	3,278.20 (1.87)	50	15.25	11.56–20.12
Adalimumab (3,271)	7,835.17 (1.97)	108	13.78	11.41–16.64
Ustekinumab (994)	2,256.44 (2.00)	34	15.07	10.77–21.09
Lower respiratory tract infections				
Non-biologics	—	27	4.21	2.88–6.13
Methotrexate	—	14	4.09	2.42–6.91
Etanercept	—	18	5.49	3.46–8.71
Adalimumab	—	31	3.96	2.78–5.63
Ustekinumab	—	12	5.32	3.02–9.36
Skin and soft tissue infections				
Non-biologics	—	22	3.43	2.26–5.20
Methotrexate	—	10	2.92	1.57–5.43
Etanercept	—	12	3.66	2.08–6.45
Adalimumab	—	19	2.42	1.55–3.80
Ustekinumab	—	8	3.55	1.77–7.09

### Crude incidence rates for serious infections overall

The incidence rate for serious infections per 1,000 person-years in the non-biologic cohort was 14.18 (95% confidence interval [CI] = 11.54–17.41), with the incidence rate for the methotrexate-only cohort at 11.98 (95% CI = 8.82–16.27). The crude incidence rates per 1,000 person-years in the biologic cohorts were etanercept, 15.25 (95% CI = 11.56–20.11); adalimumab, 13.78 (95% CI = 11.41–16.64); and ustekinumab, 15.07 (95% CI = 10.77–21.09).

### Nature of the serious infections

The most common Medical Dictionary for Regulatory Activities (i.e., MedDRA) high level term-coded serious infections experienced by participants receiving either non-biologic systemic therapy or biologic therapies were lower respiratory tract infections, followed by skin and soft tissue infections and urinary tract infections (see Supplementary Table S2 online). The crude incidence rates for serious lower respiratory tract infections and skin/soft tissue infections are presented in Table 2. Where the organism responsible for the serious infection was given or identified, the most common MedDRA high level term code was staphylococcal infections with 13 events, followed by streptococcal infections with 10 events (see Supplementary Table S3 online). There were five tuberculous infections and four herpes viral infections (herpes zoster infections). The median hospital inpatient stay ranged from 3 days (IQR = 6.0) for non-biologic therapies to 5 days for etanercept (IQR = 8.0) and adalimumab (IQR = 11.0) (see Supplementary Table S4 online). Seven participants died within 30 days of the serious infection in the non-biologic cohort; the 30-day mortality rate for etanercept was less than 5, and there were no deaths within 30 days of the event for adalimumab or ustekinumab.

### Propensity score-weighted models for the risk of serious infections

The inverse probability treatment-weighted multinomial model involving the non-biologic, etanercept, adalimumab, and ustekinumab cohorts achieved good balance, removing expected bias for most of the variables (see Supplementary Table S5 online), suggesting a reduction of confounding from these variables.

No biologic therapy showed a statistically significant increase in the risk of serious infection compared with non-biologic systemic therapies, although the risk estimate for etanercept (hazard ratio [HR] = 1.10, 95% CI = 0.75–1.60) was above 1 (Table 3). The proportionality assumption was met for etanercept and adalimumab but not for ustekinumab in the multinomial model.

**Table 3:** Crude Cox proportional hazards model and adjusted model using inverse probability treatment weighting by propensity score, showing hazard ratios from a multinomial model involving etanercept, adalimumab, and ustekinumab versus non-biologic therapy^1^

	Etanercept	Adalimumab	Ustekinumab	All Biologics
Comparison against all non-biologic systemic therapies, hazard ratios (95% confidence intervals)				
Crude	1.11 (0.79–1.57)	0.98 (0.74–1.29)	1.04 (0.70–1.54)	1.02 (0.80–1.31)
Adjusted	1.10 (0.75–1.60)	0.93 (0.69–1.26)	0.92 (0.60–1.41)	0.96 (0.73–1.27)
Concomitant immunosuppressants	1.05 (0.67–1.64)			1.09 (0.70–1.68)
Adjusted 0–6 months	*—*	*—*	2.18 (0.95–5.01)	*—*
Adjusted 6–12 months	*—*	*—*	1.20 (0.51–2.81)	*—*
Adjusted 1–2 years	*—*	*—*	0.73 (0.35–1.53)	*—*
Comparison against methotrexate				
Crude	1.37 (0.90–2.07)	1.19 (0.83–1.71)	1.26 (0.80–1.99)	1.31 (0.94–1.84)
Adjusted	1.47 (0.95–2.28)	1.26 (0.86–1.84)	1.22 (0.75–1.99)	1.29 (0.90–1.85)
Concomitant immunosuppressants	1.00 (0.64–1.57)			1.04 (0.67–1.62)

1 Exposure time with concomitant (methotrexate, cyclosporine, fumaric acid esters, hydroxycarbamide) immunosuppressive medication use is adjusted for, with exposure time to two immunosuppressive therapies classed as concomitant in the non-biologic cohort.

Analysis split by a priori defined follow-up time found a nonstatistically significant increase in risk of serious infection in the first 6 months compared with non-biologic therapies for ustekinumab (HR = 2.18, 95% CI = 0.95–5.01, Table 3) and a nonstatistically significant decrease between 1 year and 2 years of treatment (HR = 0.73, 95% CI = 0.35–1.53).

The use of concomitant immunosuppressive therapy was not associated with an increase in the risk of serious infection (HR = 1.09, 95% CI = 0.70–1.68).

### Sensitivity analyses

Analysis on a restricted cohort of entry year after 2009, the time period when all of the biologic therapies were available to be prescribed, found that the incidence rates and HRs were similar to the figures reported in the main analysis (see Supplementary Tables S6 and S7 online). The sensitivity analysis using methotrexate as the comparator found no statistically significant difference in the risk for serious infections for etanercept, adalimumab, and ustekinumab, although the effect estimates were higher (Table 3). The sensitivity analysis using a combined biologic cohort also did not show a statistically significant difference between the biologic and non-biologic or methotrexate therapies (Table 3).

The same inverse probability treatment-weighted multinomial model involving the non-biologic therapies, etanercept, adalimumab, and ustekinumab was rerun using the respective biologic therapies as the comparator instead of the non-biologic cohort, and there was no significant statistical difference found between the biologic therapies (see Supplementary Table S8 online). The sensitivity analysis models involving methotrexate and the different biologic therapies as the comparator did not violate the proportionality assumption.

## Discussion

We did not observe any statistically significant increases in the risk of serious infection for any biologic therapy versus non-biologic systemic therapies in patients with psoriasis. We show that etanercept, adalimumab, and ustekinumab have no significant differential risk of serious infection. In terms of the clinical relevance of the precision of our estimated risk, we were able to rule out a 1.6-fold increase in serious infection risk for etanercept and rule out a lower serious infection risk for adalimumab and ustekinumab over non-biologic systemic therapies. We found that the relative risk of serious infection between ustekinumab and the non-biologic systemic therapies was not constant over time.

We did not observe any association between the use of concomitant immunosuppressive therapy and increase in the risk of serious infection. However, the propensity score method balances the baseline characteristics and not time-varying factors, and hence it cannot deal adequately with confounding by indication for the use of concomitant immunosuppressive therapy. This estimated result should therefore be interpreted with caution.

### Strengths and limitations

The major strengths of this study are the prospective cohort study design, the large sample size, fully industry-independent data analysis, and the participation of multiple dermatology centers (153) in the UK and Republic of Ireland. To our knowledge, this study is the largest single registry cohort study assessing the serious infection risk of first-line biologic therapies for the treatment of psoriasis to date. Detailed data capture allowed for the inclusion of numerous covariates relevant to the risk of infection in the propensity score.

The accuracy of detailed information about the infection is dependent on information from the recruiting dermatology center. Recall bias may occur with patient-reported characteristics; this is likely to be nondifferential between the comparator treatments. Selection bias introduced by nonrandomization is controlled for by propensity score weighting for the variables that were available, but variables that were not measured and not known to be associated with the exposure or the outcome could have potentially introduced residual confounding. For example, we were not able to adjust for previous serious infection within the past year.

We included a large cohort of patients receiving methotrexate, the most prevalent non-biologic systemic therapy in BADBIR, as a single non-biologic comparator to maximize interpretability and comparison against published literature. We also used the non-biologic systemic therapies as a grouped comparator to increase the precision of the estimated risk of serious infection of the biologic therapies, which are reflected in the tighter confidence intervals compared with the methotrexate-only comparator estimates.

### Comparisons with other studies and important differences in results

We found that the distribution of the site of common infections, which included lower respiratory tract infections, skin or soft tissue infections, urinary tract infections, and abdominal infections, were similar between the non-biologic and the biologic therapies studied. This is similar to the serious infections reported in a large Dutch study of patients with psoriasis, most of whom were not receiving biologic therapies (Wakkee et al., 2011), and the results from the Psoriasis Longitudinal Assessment and Registry (PSOLAR), a large, single pharmaceutical company-sponsored study based mainly in the US and Europe, which reported pneumonia and cellulitis as the two most common serious infections in patients with psoriasis receiving non-biologic and biologic therapies (Kalb et al., 2015).

There are large variations in the reported crude incidence rate of serious infection in patients with psoriasis on biologic therapies. Our reported incidence rates for etanercept and adalimumab are similar to those reported by the PSOLAR registry (Kalb et al., 2015) and are similar to that reported for TNFIs overall in the Psocare Italian registry (Garcia-Doval et al., 2017a). Our incidence rate for serious infections with ustekinumab is higher than the PSOLAR (ustekinumab is the drug marketed by the sole sponsor of the registry) and Spanish Registry of Adverse Events from Biological Therapy in Psoriasis (i.e., BIOBADADERM) rates (Davila-Seijo et al., 2017): (BADBIR, 15.1; PSOLAR, 8.3; Spanish Registry of Adverse Events from Biological Therapy in Psoriasis, 5.9 per 1,000 person-years). These variations are to be expected, given the differences in the health care systems around the world and therefore propensity for hospitalization, a defining factor for the classification of an infection as serious. Different national clinical guidelines and funding/reimbursement arrangements may also introduce channeling effects toward certain treatments.

We found similar results to those of the PSOnet collaboration, a network of European registries of patients with psoriasis, which reported no increased risk of serious infection with TNFIs compared with a cohort receiving either acitretin, methotrexate, or cyclosporine in a meta-analysis of psoriasis treatment registries across Europe. A smaller study from Spanish Registry of Adverse Events from Biological Therapy in Psoriasis, with a focus on nonserious infections, showed no statistically significant increased risk for serious infections with TNFIs or ustekinumab versus methotrexate (Davila-Seijo et al., 2017). In contrast, an increased risk of serious infection with adalimumab was reported compared with acitretin or phototherapy in the PSOLAR registry, with similar results reported in a sensitivity analysis including methotrexate in the comparator cohort (Kalb et al., 2015).

These observational studies have different specific limitations that impede clinical interpretation. The merger of exposure to three different TNFIs (with different molecular structures, induction regimens, dosing schedule, and drug administration) into one cohort masks important distinctions between the drugs, thereby limiting the ability to inform patient choice for any particular biologic therapy (Garcia-Doval et al., 2017a). The Spanish registry lacked power to investigate serious infections (Davila-Seijo et al., 2017), and the study from the PSOLAR registry did not measure important covariates (e.g., Psoriasis Area and Severity Index, the tool used for measuring disease severity in clinical trials for psoriasis, and concomitant use of immunosuppressants), which may have led to significant residual confounding (Kalb et al., 2015) and excluded important comparators, such as cyclosporine as an alternative treatment for patients with severe psoriasis in the real world.

We believe that the use of a Cox regression survival analysis model avoids unrealistic assumptions of a constant rate and independent recurrent events that the use of a Poisson model would introduce. Only 35 of 283 (12%) of participants suffered from recurrent events, and on the balance we chose to restrict our outcome to the first serious infection to maximize interpretability with little loss in statistical power.

In contrast with our findings, TNFIs have been shown to be associated with serious infections in rheumatoid arthritis and in a time-dependent manner, with relative risks between 1.2 and 1.8 (Askling et al., 2007, Galloway et al., 2011, Strangfeld et al., 2011). However, a comparison of data from psoriasis and rheumatoid arthritis patient registries found that psoriasis patients were associated with approximately half the risk of serious adverse events (Garcia-Doval et al., 2017b); thus, safety data cannot be directly extrapolated between these two patient populations. Use of concomitant immunosuppressants, particularly systemic corticosteroids, is greater in the rheumatoid arthritis population compared with the psoriasis population and may drive some of this increased risk (Askling et al., 2007, Galloway et al., 2011, Strangfeld et al., 2011).

### Implications for patients, clinicians, and policymakers

Our results suggest that the risk of serious infection should not be a key discriminator for patients and clinicians when choosing between non-biologic systemic therapies, etanercept, adalimumab, and ustekinumab. The recently updated British Association of Dermatologists guideline for biologic therapies in psoriasis, widely used by dermatologists around the world, recommends adalimumab and ustekinumab as first-line biologic therapies (along with secukinumab) and relegates etanercept to use as a second-line biologic therapy (Smith et al., 2017). This decision was based predominantly on evidence for efficacy from clinical trials (Jabbar-Lopez et al., 2017). There is a perception that etanercept has a lower risk of serious infection than other biologics based on lower rates of tuberculosis, extrapolation from the rheumatoid arthritis literature (Galloway et al., 2011), and the general assumption that a less efficacious tumor necrosis factor inhibitors would be safer. Our results further reinforce the treatment hierarchy as suggested by the British Association of Dermatologists guidelines. It was reassuring that the distribution of types of infections was similar between the biologic and non-biologic cohorts. Furthermore, no signal emerged for any particular type of serious infection associated with biologic therapies.

### Future research opportunities

There were low rates of 30-day mortality due to serious infection for the etanercept and non-biologic cohorts, and there were no deaths in either the adalimumab or ustekinumab cohorts. This may be a result of selection bias, with channeling of patients with more comorbidities and hence higher risk of death risk to the treatments with a lower perceived risk. Investigation within a larger cohort and with a longer follow-up period is welcomed to investigate whether there is any association between infection-related 30-day mortality and the different therapies.

### Summary

We did not find a statistically significant higher relative risk of serious infections for etanercept, adalimumab, and ustekinumab compared with non-biologic therapies for patients with psoriasis. There was no difference in the risk of serious infections between etanercept, adalimumab, and ustekinumab. The risk of serious infection, therefore, should not be a primary concern for patients and clinicians when deciding between non-biologic systemic therapies or these three biologic therapies for psoriasis. Health care professionals should be equally vigilant for serious infections when managing patients with psoriasis who are receiving either systemic non-biologic or biologic therapies.

## Materials and Methods

BADBIR was approved in March 2007 by the National Health Service Research Ethics Committee North West England, reference 07/MRE08/9. All subjects gave written informed consent for their participation in the registry.

BADBIR is a large, ongoing, prospective pharmacovigilance registry of psoriasis patients that was established in 2007 in the UK and Ireland to compare the safety of biologic therapies against non-biologic systemic therapies. Establishing the risk of serious infections was a prespecified primary aim of the registry. The design of BADBIR (Burden et al., 2012) and the baseline patient characteristics (Iskandar et al., 2015) have been published previously. In England, the National Institute for Health and Care Excellence recommends that all patients with psoriasis receiving biologic therapies should be registered on BADBIR. Subjects were selected in a data snapshot from October 2016.

### Baseline assessment

Baseline data were collected before or during the initial 6 months of treatment. Drug, clinical, and comorbid history along with anthropometric data were collected by a health care professional using a web-administered questionnaire, and lifestyle factors were collected by a patient completed questionnaire.

### Follow-up assessments

Data from patients were collected every 6 months for the first 3 years, then annually thereafter to 10 years. Follow-up data were collected and entered onto a web-based system contemporaneously. Specific information about serious infections was collected, including descriptions of events, hospitalization, and start and stop dates. Patient death information was collected from BADBIR and validated using the Office of National Statistics mortality records. Additional external data on serious infections in Wales was provided by the National Health Service Welsh Informatics Service and linked onto BADBIR (12 additional events obtained). Adverse events were classified using the Medical Dictionary for Regulatory Activities system.

### Data analysis

The main inclusion criteria for this study were biologic-naïve patients with chronic plaque psoriasis starting either a licensed biologic therapy for psoriasis (i.e., etanercept, adalimumab, and ustekinumab), who were recruited into the biologic cohort, and biologic-naïve patients with chronic plaque psoriasis receiving either acitretin, psoralen-UVA, cyclosporine, fumaric acid esters, methotrexate, and hydroxycarbamide, who were recruited into the non-biologic systemic cohort. Secukinumab was excluded because there were not enough patients receiving the therapy for meaningful analysis at data lock (n = 12) (Figure 1). Infliximab was considered to be significantly different from both the non-biologic cohort and the other three biologic therapies, given the higher prescription criteria (Psoriasis Area and Severity Score ≥ 20 and Dermatology Life Quality Index > 18) stipulated by the National Institute for Health and Care Excellence, and power to study infliximab was also limited (n = 105). An initial exploratory analysis also confirmed these findings, because the cohort receiving infliximab had a higher disease severity and more comorbidities compared with the non-biologic and other biologic cohorts (see Supplementary Table S9 online).

Patients were included if follow-up data were available. No biosimilar drugs were included in this study. Patients in the biologic cohort contributed follow-up time from the first dose of the drug until the first event of the following: serious infection, discontinuation of treatment due to other reasons, last registered follow-up, switch to other biologic therapy, or death. Patients in the non-biologic cohort contributed follow-up time from first dose of the index drug until the first event of all of the mentioned events but were censored at the end of the last alternative non-biologic therapy. Patients who switched from the non-biologic therapy cohort to start receiving a biologic therapy contributed follow-up time to both cohorts. Second or subsequent lines of biologic therapy were not eligible for this study.

A *serious infection* was defined as any infection that was associated with or prolonged hospitalization, required the use of intravenous antimicrobial therapy, or led to death. The inclusion of intravenous antimicrobial therapy use is a pragmatic addition to the International Conference on Harmonisation definition of *serious adverse event specific to infections.* The events were validated by separate review from two clinicians (ZZNY, RBW) against these criteria, and discrepancies (n = 41) were resolved through discussion. A clinical specialist relevant to the specific type of infection was consulted in cases for which there was uncertainty. The first serious infection was included for analysis in this study, with a risk window period of 90 days after cessation of treatment applied for the attribution of the event to the drug (Galloway et al., 2011).

The licensed dosing regimens for the biologic therapies are as follows: etanercept 50 mg once weekly by subcutaneous injection, adalimumab 40 mg every other week starting 1 week after an initial dose of 80 mg by subcutaneous injection, and ustekinumab 45 mg (90 mg for patients of 100 kg or greater) initially, at week 4, week 12, and every 12 weeks thereafter by subcutaneous injection administered by health care professionals. The impact of alternate dosing regimens was not analyzed because the proportion of patients using cumulative doses different from the licensed dosing regimens is low in the UK (<15% [Iskandar et al., 2017]), and National Institute for Health and Care Excellence-approved dosing regimen is according to the license. Within the biologic cohort, the number of person-years receiving doses outside the license was too low to make statistical inferences for the effect of dosing regimen on the risk of serious infection.

Sample size was based on detecting or ruling out a 2-fold increase in serious infection risk as compared with the non-biologic systemic cohort, which was considered a clinically relevant difference by consensus of the BADBIR data analysis committee.

### Primary analyses

To provide a description of the rates of serious infections, crude incidence rates for each drug in the biologic cohort and the non-biologic cohort were calculated as the number of events per 1,000 patient-years of follow-up. Survival modeling with Cox proportional hazards was used to compare event rates and estimate the effect of each exposure on the risk of serious infections.

A priori potential confounders to include in the multivariate analysis were based on expert opinion and a literature review (Yiu et al., 2016). These were age, sex, body mass index, waist circumference, alcohol use, disease severity (Psoriasis Area and Severity Index), concomitant inflammatory arthritis including psoriatic arthritis and ankylosing spondylitis, smoking, diabetes, chronic obstructive pulmonary disease, asthma, immunodeficiency syndromes, and concomitant immunosuppressants. The total number of measured comorbidities was included as a separate covariate as a proxy for patient frailty. Conditions under immunodeficiency syndromes include HIV infection and lymphopenia. Body mass index was presented as a categorical variable to ease data description in Table 1 but was kept as a continuous variable in the statistical models. Adjustment for the baseline potential confounders was performed using a propensity score model. A probability score for having the treatment was derived from a multinomial logistic regression model based on the baseline-relevant covariates listed. The use of propensity score adjustment has various advantages over multivariable regression models, in particular the ability to check the balance of measured confounders between the comparator cohorts, and improving estimation when outcome is rare by allowing for multiple covariates (Glynn et al., 2006).

Inverse probability treatment weighting, where the treatments were weighted for the distribution of the propensity score in the whole model cohort, was then performed using propensity score probabilities in both models. Balance between groups after weighting was assessed using expected bias from a logistic regression model estimating the effect of the variable on serious infection. Improvement in balance was achieved by an iterative process of fitting interaction terms involving the least balanced variables.

Concomitant therapies that were considered to be immunosuppressants were methotrexate, cyclosporine, fumaric acid esters, and hydroxycarbamide. Concomitant immunosuppressants (defined as the exposure period to more than one immunosuppressant in the non-biologic cohort) were treated exceptionally as time-varying covariates, allowing for the time on and off these drugs throughout follow-up.

Missing data (see Supplementary Table S10 online) were imputed in a multiple imputation model of 20 cycles to reduce bias (Sterne et al., 2009). We used multiple imputation to account for missing data for the potential confounders, because this preserves the variability and uncertainty of the missing data and avoids the loss of power and bias that alternative ad hoc methods, such as a complete case analysis, may introduce. Propensity likelihood scores were calculated in each imputed dataset and combined after regression modeling using Rubin’s rules. A key assumption for the Cox regression is the proportionality assumption, where the relative risk between the comparators is constant over time. Formal testing for proportionality using Schoenfeld residuals in the Cox regression model was performed in five extracted imputed datasets, and where the proportionality assumption did not hold a time-stratified analysis using the prespecified time points of 0–6 months, 6–12 months, and 12–24 months of follow-up, which were the designated follow-up data reporting time points, was performed.

### Secondary analyses

A priori planned sensitivity analyses included methotrexate users as the comparator cohort as the most common systemic non-biologic in use, combining all three biologic cohorts as one cohort to compare against all non-biologic systemic therapies and methotrexate, and restriction to patients starting treatment on or after 2009 (when the three biologic therapies were available for prescription at the same time). Descriptive analysis was performed on soft tissue and skin infections, and lower respiratory tract infections as these were identified as common infections associated with patients receiving biologic therapies, but the lower number of events did not allow for meaningful multivariate analysis of relative risks (see Supplementary Table S2).

The actual number of events for any data point involving the individual biologic therapies that has fewer than five events in the descriptive analysis has been removed for the protection of participant confidentiality. All analyses were performed using Stata 14 (StataCorp, College Station, TX).

## ORCID

Zenas ZN Yiu: http://orcid.org/0000-0002-1831-074X

## Conflict of Interest

ADO has received consultation fees from Janssen, Eli-Lilly, Amgen, and LEO; speaker fees from Novartis; and unrestricted research support from Merck, Pfizer, Janssen, and Abbvie.

CEMG is a National Institute for Health Research Senior Investigator. CEMG reports grants from the National Institute for Health Research during the conduct of the study; grants and personal fees from GlaxoSmithKline, AbbVie, Lilly, Novartis, Pfizer, Janssen, LEO, and Celgene; grants from Sandoz; personal fees from Sun Pharmaceuticals, UCB Pharma, and Almirall; and grants and personal fees from outside the submitted work.

CHS has received research grants from Abbvie, Pfizer, Novartis, GlaxoSmithKline, Roche, and Regeneron.

DMA was supported by a research grant from Abbvie and has had personal fees from Pfizer and GlaxoSmithKline.

NJR has received honoraria, travel support, and/or research grants (Newcastle University) from Abbvie, Amgen, AstraZeneca, Bristol-Myers Squibb, Celgene, Genentech, Janssen, Leo-Pharma Research Foundation, Novartis, Pfizer, and Stiefel GSK.

RBW has received research grants from Abbvie, Pfizer, Novartis, and Leo and reports personal fees from AbbVie, Amgen, Boehringer Ingelheim Pharma, Celgene, Janssen-Cilag, Leo, Lilly, Novartis, Pfizer, and Xenoport outside the submitted work.

SW reports personal fees from Abbvie and Novartis outside the submitted work.

ZZNY has received nonfinancial support from Novartis outside the submitted work.

ML and RM have no conflicts of interests to disclose.
