# Supplementary material for: Risk of Serious Infection in Patients with Psoriasis Receiving Biologic Therapies: A Prospective Cohort Study from the British Association of Dermatologists Biologic Interventions Register (BADBIR)
Source: J Invest Dermatol. 2018 Mar;138(3):534–41. doi: 10.1016/j.jid.2017.10.005 (PMC5832757; doi:10.1016/j.jid.2017.10.005)
Supplement: Supplementary Tables S1–S10 [file mmc1.pdf]

Supplementary Table S1 – Proportion of participants lost to follow-up at each data collection timepoint, excluding participants who have been censored for the end of follow-up.

	**Non-biologics**	**Etanercept**	**Adalimumab**	**Ustekinumab**
**0-6 months**				
Adverse events	136 (4.0%)	27 (2.0%)	71 (2.2%)	10 (1.0%)
Death	0 (0.0%)	1 (0.1%)	1 (0.0%)	0 (0.0%)
Ineffectiveness	113 (3.3%)	139 (10.3%)	130 (4.0%)	5 (0.5%)
Remission	23 (0.7%)	4 (0.3%)	1 (0.0%)	1 (0.1%)
Other	148 (4.3%)	25 (1.8%)	59 (1.8%)	9 (0.9%)
Missing	7 (0.2%)	4 (0.3%)	4 (0.1%)	2 (0.2%)
**TOTAL:**	**427 (12.5%)**	**200 (14.8%)**	**266 (8.1%)**	**27 (2.7%)**
**6-12 months**				
Adverse events	104 (3.0%)	28 (2.1%)	80 (2.4%)	12 (1.2%)
Death	0 (0.0%)	1 (0.1%)	2 (0.1%)	0 (0.0%)
Ineffectiveness	183 (5.3%)	169 (12.5%)	264 (8.1%)	22 (2.2%)
Remission	15 (0.4%)	4 (0.3%)	26 (0.8%)	0 (0.0%)
Other	205 (6.0%)	37 (2.7%)	69 (2.1%)	12 (1.2%)
Missing	9 (0.3%)	9 (0.7%)	3 (0.1%)	0 (0.0%)
**TOTAL:**	**516 (15.1%)**	**248 (18.3%)**	**444 (13.6%)**	**46 (4.6%)**
**12-24 months**				
Adverse events	180 (5.3%)	23 (1.7%)	62 (1.9%)	19 (1.9%)
Death	4 (0.1%)	0 (0.0%)	2 (0.1%)	2 (0.2%)
Ineffectiveness	259 (7.6%)	149 (11.0%)	178 (5.4%)	29 (2.9%)
Remission	36 (1.1%)	4 (0.3%)	20 (0.6%)	4 (0.4%)
Other	290 (8.5%)	35 (2.6%)	73 (2.2%)	12 (1.2%)
Missing	21 (0.6%)	3 (0.2%)	6 (0.2%)	1 (0.1%)
**TOTAL:**	**790 (23.1%)**	**214 (15.8%)**	**341 (10.4%)**	**67 (6.7%)**
**24 months or more**				
Adverse events	230 (6.7%)	29 (2.1%)	34 (1.0%)	6 (0.6%)
Death	5 (0.1%)	4 (0.3%)	4 (0.1%)	2 (0.2%)
Ineffectiveness	331 (9.7%)	162 (12.0%)	91 (2.8%)	25 (2.5%)
Remission	57 (1.7%)	2 (0.1%)	3 (0.1%)	1 (0.1%)
Other	427 (12.5%)	37 (2.7%)	67 (2.0%)	16 (1.6%)
Missing	25 (0.7%)	2 (0.1%)	7 (0.2%)	3 (0.3%)
**TOTAL:**	**1075 (31.4%)**	**236 (17.5%)**	**206 (6.3%)**	**53 (5.3%)**

Supplementary Table S2 - Location type of common first serious infections as coded by the MEDDRA High Level Terms (HLT); percentage proportion of type of serious infection out of participants on each drug

MEDDRA HLT Codes	Non-biologics	Etanercept	Adalimumab	Ustekinumab
Lower respiratory tract infections and lung infections	27 (0.8%)	18 (1.3%)	31 (1.0%)	12 (1.2%)
Skin structures and soft tissue infections	22 (0.7%)	12 (1.1%)	19 (0.7%)	8 (1.0%)
Urinary tract infections	8 (0.2%)	9 (0.7%)	10 (0.3%)	5 (0.5%)
Abdominal and gastrointestinal infections	8 (0.2%)	<5	6 (0.2%)	<5
Upper respiratory tract infections	7 (0.2%)	<5	10 (0.3%)	<5
Sepsis, bacteraemia, viraemia and fungaemia	<5	6 (0.4%)	8 (0.2%)	<5
Bone and joint infections	<5	<5	<5	<5

Supplementary Table S3 - Organism type of first serious infections as coded by the MEDDRA High Level Terms (HLT) for the all therapies combined.

MEDDRA HLT Codes	Number of infections
**Bacterial**	
Campylobacter infections	2
Escherichia infections	7
Haemophilus infections	2
Klebsiella infections	1
Legionella infections	2
Moraxella infections	1
Neisseria infections	1
Pseudomonas infections	1
Staphylococcal infections	13
Streptococcal infections	10
Tuberculous infections	5
**Viral**	
Adenoviral infections	1
Herpes viral infections	4
Hepatitis viral infections	1
Retroviral infections	1
**Others**	
Amoebic infections	1
Candida infections	1

Supplementary Table S4 – Length of hospital stay for the first serious infection, and the associated 30 day mortality.

	Median stay in hospital (IQR) - days	Number at risk and number of serious infections	30 day mortality for first serious infection
**Non-biologic**	3.0 (6.0)	3421; 91	7
**Etanercept**	5.0 (8.0)	1352; 50	<5
**Adalimumab**	5.0 (11.0)	3271; 108	0
**Ustekinumab**	4.0 (6.0)	994; 34	0

Supplementary Table S5 – The baseline characteristics/variables of the different drug cohorts after inverse probability treatment weighting by propensity score to the respective biologic therapy (multi-nomial logistic regression model involving non-biologic, etanercept, adalimumab and ustekinumab).

Characteristic	Non-biologic	Etanercept	Expected bias in β before weighting	Expected bias in β after weighting	Adalimumab	Expected bias in β before weighting	Expected bias in β after weighting	Ustekinumab	Expected bias in β before weighting	Expected bias in β after weighting
Age	45.01	45.21	1.4%	0.3%	44.87	0.2%	-0.2%	45.06	2.0%	0.1%
Waist	100.92	101.00	-0.3%	-0.0%	100.93	-0.3%	-0.0%	100.56	-0.9%	0.1%
BMI	30.60	30.60	0.2%	-0.0%	30.59	0.2%	-0.0%	30.42	0.9%	-0.1%
Alcohol (units per week)	8.31	8.35	1.0%	0.0%	8.18	0.4%	-0.1%	8.22	-0.0%	-0.0%
PASI	15.98	16.01	-0.5%	0.0%	15.95	0.0%	-0.0%	16.10	0.2%	0.1%
Disease duration	21.04	20.99	-0.8%	0.0%	20.87	-0.6%	0.0%	20.77	-0.6%	0.1%
Smoking (no. cigarettes per day)	4.13	4.05	-0.7%	-0.1%	4.13	-0.9%	0.0%	4.06	-0.9%	-0.1%
Number of comorbidities	1.37	1.36	8.3%	-0.2%	1.35	5.1%	-0.3%	1.35	9.5%	-0.4%
Female gender	41.8%	41.6%	-0.2%	-0.0%	41.7%	-0.4%	-0.0%	42.3%	-0.7%	0.1%
Hypertension	22.7%	22.5%	0.0%	-0.0%	22.7%	0.0%	0.0%	22.8%	0.0%	0.0%
Asthma	10.5%	10.7%	-0.1%	0.1%	10.6%	-0.0%	0.0%	10.7%	0.5%	0.0%
COPD	1.8%	1.7%	-0.3%	-0.0%	1.9%	-0.3%	0.0%	1.7%	0.2%	-0.0%
Previous TB	1.0%	0.8%	-0.6%	0.1%	1.0%	-0.2%	-0.0%	0.9%	-0.1%	0.1%
Diabetes	8.4%	8.8%	-0.4%	-0.1%	8.4%	-0.0%	0.0%	8.6%	-0.6%	-0.0%
Dyslipidaemia	10.4%	10.6%	-0.8%	-0.1%	10.0%	-0.6%	0.2%	9.9%	-1.6%	0.2%
Inflammatory arthritis	18.4%	18.4%	0.2%	0.0%	18.3%	0.3%	-0.0%	18.2%	0.1%	-0.0%
Immunodeficiency syndromes	0.2%	0.1%	0.0%	0.0%	0.2%	0.0%	0.0%	0.2%	0.0%	0.0%

Supplementary Table S6 - Crude incidence rates of first serious infection – entry year on or after 2009

Treatment	N (9032)	Person-time (years)	Infections	Rate (per 1000 person-years)	95% Confidence Interval (person-years)
**Non-biologics**	3390	6116.95	91	14.88	12.11, 18.27
**Etanercept**	1234	2850.93	48	16.84	12.69, 22.34
**Adalimumab**	3252	7722.40	103	13.34	11.00, 16.18
**Ustekinumab**	994	2256.44	34	15.07	10.77, 21.09

Supplementary Table S7 - Sensitivity analyses of restriction to cohort with entry year above 2009 when etanercept, adalimumab and ustekinumab were all available. Cox proportional hazards models using inverse probability treatment weighting by propensity score are presented, showing effect estimates from the multi-nomial model involving Etanercept, Adalimumab and Ustekinumab against Non-biologic therapy; Adjustment was made in the model for the time-varying covariate of concomitant immunosuppressive (MTX for biologic therapies, CYA, FAE, HYC).

Time period	Etanercept	Adalimumab	Ustekinumab
**Overall Hazard Ratio (95% confidence intervals)**	1.16 (0.79,1.69)	0.87 (0.64,1.19)	0.89 (0.58,1.36)

Supplementary Table S8 – The propensity score weighted Cox proportional hazard models in Table 3 were rerun using the different biologic therapies as the comparator instead of the non-biologic cohort.

	Non-biologics	Etanercept	Adalimumab	Ustekinumab
**Etanercept as comparator**				
Crude Hazard Ratio (HR; 95% Confidence Interval)	0.90 (0.63,1.27)	Ref	0.88 (0.63,1.23)	0.93 (0.60,1.45)
Adjusted HR	0.91 (0.63,1.33)	Ref	0.85 (0.60,1.20)	0.84 (0.53,1.34)
**Adalimumab as comparator**				
Crude HR	1.02 (0.77,1.35)	1.14 (0.82,1.60)	Ref	1.07 (0.72,1.57)
Adjusted HR	1.07 (0.79,1.45)	1.18 (0.83,1.67)	Ref	0.99 (0.66,1.49)
**Ustekinumab as comparator**				
Crude HR	0.96 (0.65,1.42)	1.07 (0.69,1.66)	0.94 (0.64,1.38)	Ref
Adjusted HR	1.09 (0.71,1.66)	1.19 (0.75,1.90)	1.01 (0.67,1.53)	Ref

Supplementary Table S9 – The baseline demographic and disease characteristics of the infliximab and non-biologic cohort

**Characteristics**	**Non-biologic cohort (n=3421)**	**Infliximab (n=105)**
Age (year) mean(SD)	44.6 (14.0)	46.6 (13.5)
Female	1489 (43.5%)	32 (30.5%)
Waist circumference (cm) mean(SD)	99.7 (17.1)	105.6 (19.6)
**BMI category (kg/m^2)**		
Underweight (<18.5)	43 (1.3%)	1 (1.0%)
Normal (18.5-24.9)	677 (19.8%)	19 (18.1%)
Overweight (25.0-29.9)	1071 (31.3%)	18 (17.1%)
Obese I (30.0-34.9)	735 (21.5%)	20 (19.0%)
Obese II (35.0-39.9)	345 (10.1%)	15 (14.3%)
Obese III (≥40)	279 (8.2%)	21 (20.0%)
**Comorbidities and risk factors**		
No comorbidity	1323 (38.7%)	25 (23.8%)
1-2 comorbidities	1585 (46.3%)	48 (45.7%)
3-4 comorbidities	416 (12.2%)	23 (21.9%)
≥5 comorbidities	97 (2.8%)	9 (8.6%)
Hypertension	620 (18.1%)	35 (33.3%)
Past TB	21 (0.6%)	2 (1.9%)
Diabetes Mellitus	254 (7.4%)	15 (14.3%)
Dyslipidaemia	307 (9.0%)	14 (13.3%)

Asthma	361 (10.6%)	9 (8.6%)
COPD	69 (2.0%)	1 (1.0%)
Immunodeficiency syndromes	6 (0.2%)	0 (0.0%)
Number of cigarettes smoked per day, mean (SD)	4.6 (7.7)	5.9 (8.8)
Alcohol units per week, mean (SD)	7.7 (12.1)	10.9 (23.8)
**Disease**		
Disease duration (year)<br>Median (IQR)	18.0 (18.0)	19.0 (16.0)
Baseline PASI score Median (IQR)	14.1 (7.9)	24.6 (12.3)
Inflammatory arthritis	363 (10.6%)	33 (31.4%)
**Concomitant treatments**		
Any exposure to Methotrexate during F/U	2118 (61.9%)	28 (26.7%)
Any exposure to Ciclosporin during F/U	1216 (35.6%)	6 (5.7%)
Any exposure to Fumaric Acid Esters during F/U	552 (16.1%)	2 (1.9%)
Any exposure to Hydroxycarbamide during F/U	56 (1.6%)	5 (4.8%)

*List of predefined comorbidities include: hypertension, angina, myocardial infarction, stroke, epilepsy, asthma, chronic obstructive pulmonary disease, peptic ulcer disease, chronic renal disease, liver disease, previous tuberculosis, demyelination, diabetes mellitus, impaired glucose tolerance, depression, dyslipidaemia, non-skin cancer, immunodeficiency syndromes, thyroid disease.

Supplementary Table S10 - Missing data

Variable	N	%
**Disease duration**	142	1.6%
**BMI**	692	7.7%
**Alcohol**	1023	11.3%
**Waist**	1161	12.8%
**Smoking**	1302	14.4%
**PASI**	1803	19.9%
